# Neural substrates of embodied natural beauty and social endowed beauty: An fMRI study

**DOI:** 10.1038/s41598-017-07608-8

**Published:** 2017-08-02

**Authors:** Wei Zhang, Xianyou He, Siyan Lai, Juan Wan, Shuxian Lai, Xueru Zhao, Darong Li

**Affiliations:** 1 Guangdong Key Laboratory of Mental Health and Cognitive Science, Center for Studies of Psychological Application, School of Psychology, South China Normal University, Guangzhou, P. R. China; 2 School of Architecture, Guangdong Engineering & Technology Research Center for Modern Architecture Design, State Key Laboratory of Subtropical Building Science, South China University of Technology, Guangzhou, P. R. China; 3 Key Laboratory of Chinese Learning and International Promotion of South China Normal University, Guangzhou, P. R. China; 4 Public Kindergarten of Guangzhou Government, Guangzhou, P. R. China

## Abstract

What are the neural mechanisms underlying beauty based on objective parameters and beauty based on subjective social construction? This study scanned participants with fMRI while they performed aesthetic judgments on concrete pictographs and abstract oracle bone scripts. Behavioral results showed both pictographs and oracle bone scripts were judged to be more beautiful when they referred to beautiful objects and positive social meanings, respectively. Imaging results revealed regions associated with perceptual, cognitive, emotional and reward processing were commonly activated both in beautiful judgments of pictographs and oracle bone scripts. Moreover, stronger activations of orbitofrontal cortex (OFC) and motor-related areas were found in beautiful judgments of pictographs, whereas beautiful judgments of oracle bone scripts were associated with putamen activity, implying stronger aesthetic experience and embodied approaching for beauty were elicited by the pictographs. In contrast, only visual processing areas were activated in the judgments of ugly pictographs and negative oracle bone scripts. Results provide evidence that the sense of beauty is triggered by two processes: one based on the objective parameters of stimuli (embodied natural beauty) and the other based on the subjective social construction (social endowed beauty).

## Introduction

How people perceive beauty is the most important and debated issue in aesthetic psychology and neuroaesthetics. According to aesthetic theories, a sense of beauty may be mainly affected by two factors^1^. The first is objective parameters and external morphology of concrete objects, consistent with the perspectives of Plato’s *objectivist view* of aesthetic perception^1^. Previous studies have found symmetrical human faces^2–4^, geometrical shapes^5^ and websites designs^6^, symmetrical sequences of apparent movements^7^, representational artworks^8–10^, sculptures obeying the canonical proportion of the golden ratio^1^, and paintings and polygon patterns with intermediate complexity^11, 12^, typically elicit higher aesthetic appraisal and experience, providing support of this theory that “beauty has corresponding morphological characteristics”.

An alternative theory argues that the perception of beauty is mainly based on the subjective construction of valence based on abstract social meaning and values. Prior studies have found smiling human faces^13^ with direct eye gaze^14^, people with good inner character^15–17^, and short sentences^18^ and scenes^19^ describing morally-positive actions can elicit a sense of beauty and produce enhanced activation of aesthetic reward systems due to the positive social meanings associated with these stimuli. These studies imply that aesthetic evaluation is a subjective construction and support the “what is good is beautiful” theory.

Although there has been progress in the neuroimaging of aesthetic appraisal, very little is known regarding the mental and neural mechanisms underlying these seemingly contradictory theories. Previous studies have found that ancient Chinese characters can arouse a sense of beauty due to their obvious two-dimensional graphical features^20, 21^, supporting the use of ancient Chinese characters as materials for studies of aesthetic appraisal and judgments. More importantly, ancient Chinese characters were produced using two separate systems, one in which characters referring to concrete objects were generated by outlining the shape of the object (pictographs), and another in which characters referring to abstract social meaning were developed to convey corresponding social concepts (ideographic symbol of oracle bone scripts)^22, 23^. Aesthetic qualities of pictographs and oracle bone script therefore may be based on the beauty of objective morphologies and the beauty of subjective social construction, respectively.

Following the logic of the studies examining aesthetic perception and appraisal of pictographs^20, 21^, we asked the question of whether oracle bone scripts would elicit aesthetic appraisal dependent on their abstract social meanings. That is, would oracle bone scripts that refer to positive social meanings be more likely to be judged as beautiful, and those that refer to negative social meanings be more likely to be judged as ugly? The answer to this question may contribute to identifying the mental and neural mechanisms underlying the beauty of objective morphologies and the beauty of subjective social construction.

The present functional magnetic resonance imaging (fMRI) study investigated the neural basis of aesthetic appraisal of objective morphologies and subjective social constructions when participants observed and made aesthetic judgments about ancient Chinese characters referring to concrete objects (aesthetic judgment of pictograph, AP) and abstract social meanings (aesthetic judgment of oracle bone script, AO). The effect of semantic processing during the aesthetic judgments was prevented by using pictographs and oracle bone scripts that were unfamiliar to participants. In addition, high or low luminance grey squares were used in a baseline task (square luminance judgment, SL) to control for activity in motor brain regions associated with the key responses. The neural substrates of pictograph aesthetic judgments were identified by the contrast of “AP > SL”, and the neural substrates of oracle bone script aesthetic judgments were identified by the contrast of “AO > SL”. According to our hypothesis, if pictographs and oracle bone scripts elicit variable aesthetic appraisals related to the aesthetic qualities of the referential objects and the positive degrees of the referential social meanings, respectively, then similar neural areas should be engaged in both tasks, including regions of the fusiform gyrus, occipital gyri, inferior and middle frontal gyri, anterior cingulate cortex, OFC, caudate nucleus and putamen which are associated with perceptual, cognitive, emotional and reward processing^24–26^.

Because pictographs reflect the shapes of their referential objects, they have a higher degree of visualization than oracle bone scripts referring to abstract meanings. We hypothesized that this high visualization for pictographs would activate perceptual representations, and produce relevant image schemas and visual mental images which could serve to elicit strong recurring bodily experiences^27^. These bodily experiences might result in an embodied contribution towards beauty, which can be regarded as bodily resonance for the intention of making action toward beauty^21, 28, 29^, and which should be revealed by stronger activations in motor-related regions^21, 28^.

## Results

### Behavioral results

#### Judgments of Beauty

Judgments of beauty were used as behavioral dependent variable, calculated as the rates at which pictographs or oracle bone scripts were judged to be beautiful. A 2 (type of referent: pictograph referring to concrete objects vs. oracle bone script referring to abstract social meanings) × 2 (valence of referent: positive vs. negative) repeated-measures ANOVA revealed a significant main effect of type of referent, *F* (1, 16) = 64.28, *p* < 0.001, *MSe* = 0.02, *ƞ* ^2^ = 0.80. A significant main effect of valence of referent was also found, *F* (1, 16) = 338.07, *p* < 0.001, *MSe* = 0.01, *ƞ* ^2^ = 0.96, as well as a significant interaction between type of referent and valence of referent, *F* (1, 16) = 8.83, *p* < 0.010, *MSe* = 0.01, *ƞ* ^2^ = 0.36.

The simple effect of beauty for the pictographs revealed higher ratings when pictographs referred to beautiful objects (0.73 ± 0.13) than when they referred to ugly objects (0.32 ± 0.13), *F* (1, 16) = 90.98, *p* < 0.001, *ƞ* ^2^ = 0.85. Likewise, oracle bone scripts were rated higher in beauty when they referred to positive social meanings (0.70 ± 0.12) than to negative social meanings (0.17 ± 0.08), *F* (1, 16) = 729.54, *p* < 0.001, *ƞ* ^2^ = 0.98. There was no significant difference in beauty judgment between pictographs that referred to beautiful objects and oracle bone scripts that referred to positive social meanings, *F* (1, 16) = 1.44, *p* = 0.248, *ƞ* ^2^ = 0.08. However, the rate of beautiful judgments for pictographs that referred to ugly objects was higher than that for oracle bone scripts that referred to negative social meanings, *F* (1, 16) = 39.10, *p* < 0.001, *ƞ* ^2^ = 0.71, suggesting that viewing pictographs and oracle bone scripts could differentially arouse the sense of beauty depending on the valence of their referents.

#### Post-scan ratings

The mean ratings performed post-scanning showed participants overall reported that it was difficult to guess the semantic meaning of any of the characters. Low ratings held across character and valence, including pictographs referring to beautiful objects (1.76 ± 0.32) and ugly objects (1.57 ± 0.32), and oracle bone scripts referring to positive (1.72 ± 0.45) and negative social meanings (1.39 ± 0.29). These consistently low ratings suggest that it was unlikely that participants based their aesthetic judgments of the four sets of stimuli on their semantic meanings.

### fMRI results

#### Brain regions for aesthetic judgments of pictographs with aesthetic valence

**Figure 1:**
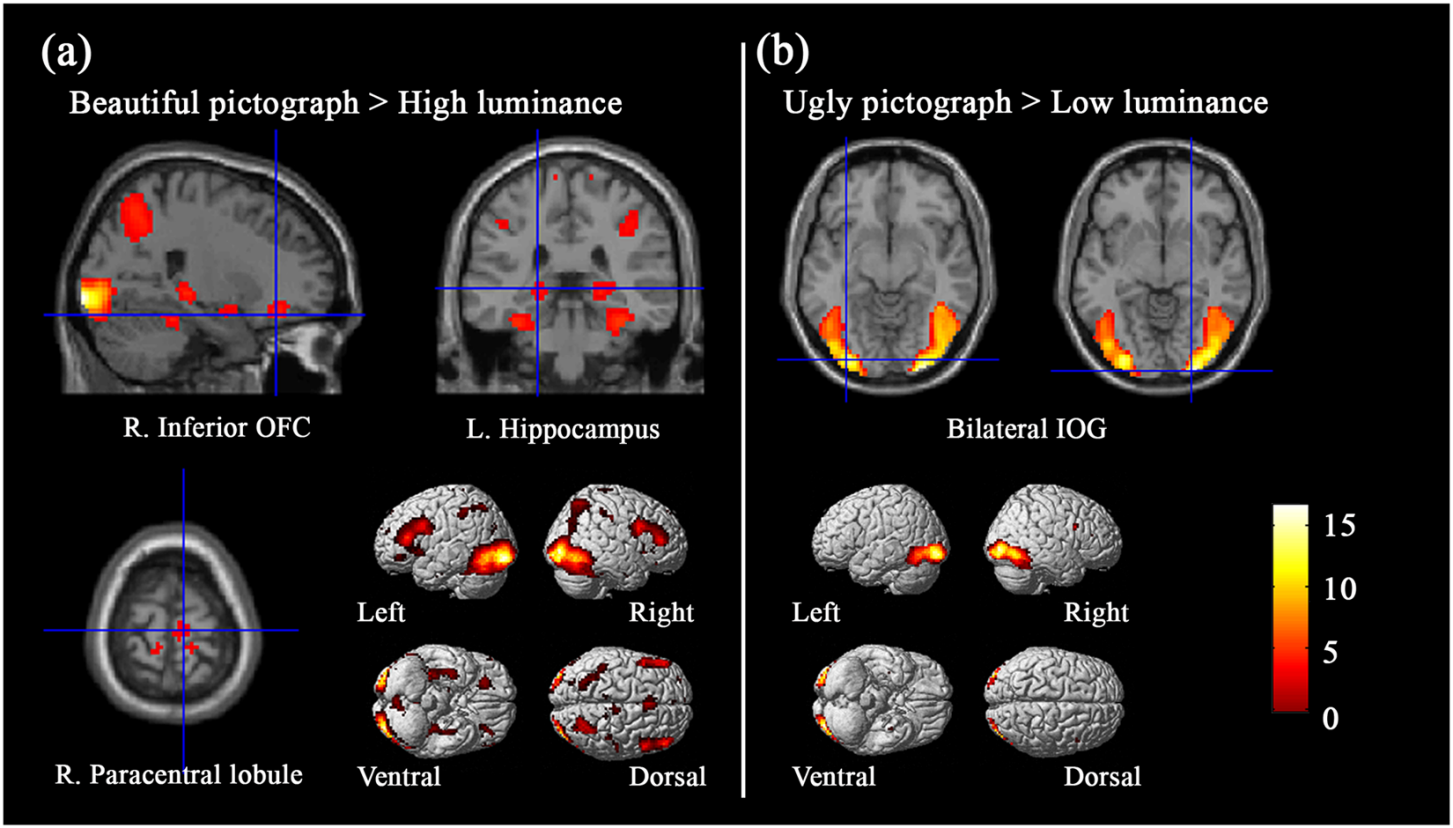
The main cerebral areas for aesthetic judgments of pictographs.

In order to identify brain regions sensitive to aesthetic valence of pictographs, the “PB > HL” and “PU > LL” contrasts were examined. Similar to the findings of Zhang *et al*.^21^, brain activity related to the contrast of “PB > HL” was found in large-scale brain networks, including the bilateral inferior occipital gyri, left middle occipital gyrus, bilateral inferior frontal gyri, left medial superior frontal gyrus, right inferior OFC, left hippocampus, left superior parietal lobule, right supramarginal gyrus extending to postcentral gyrus, and right paracentral lobule (see Table 1 and Fig. 1a). Only bilateral inferior occipital gyri were activated in the contrast of “PU > LL” (see Table 1 and Fig. 1b).

**Table 1:** Activated areas correlating with the aesthetic judgments of pictographs.

Brain regions	Hemisphere	Peak coordinates			*t*-score	Cluster size
		x	y	z		
**Beautiful pictograph > High luminance**						
Inferior occipital gyrus	L	−33	−90	−9	17.70	1736
	R	24	−99	−6	19.51	2309
Middle occipital gyrus	L	−24	−72	30	5.73	369
Inferior frontal gyrus	L	−48	30	15	7.50	827
	R	45	12	27	7.27	611
Medial superior frontal gyrus	L	−6	21	42	5.26	195
Inferior OFC	R	24	30	−18	5.23	67
Hippocampus	L	−21	−33	0	4.50	50
Superior parietal lobule	L	−24	−63	48	5.31	369
Supramarginal gyrus extending to postcentral gyrus	R	39	−30	39	4.57	77
Paracentral lobule	R	9	−24	75	4.33	32
**Ugly pictograph > Low luminance**						
Inferior occipital gyrus	L	−33	−90	−9	13.59	638
	R	24	−99	−6	14.24	763

Note: Coordinates refer to the stereotactic space of the Montreal Neurological Institute. The statistical significance refers to *p* < 0.001 at voxel level (uncorrected), *p* < 0.05 at cluster level (FWE corrected).

#### Brain regions for aesthetic judgments of oracle bone scripts with aesthetic valence

**Figure 2:**
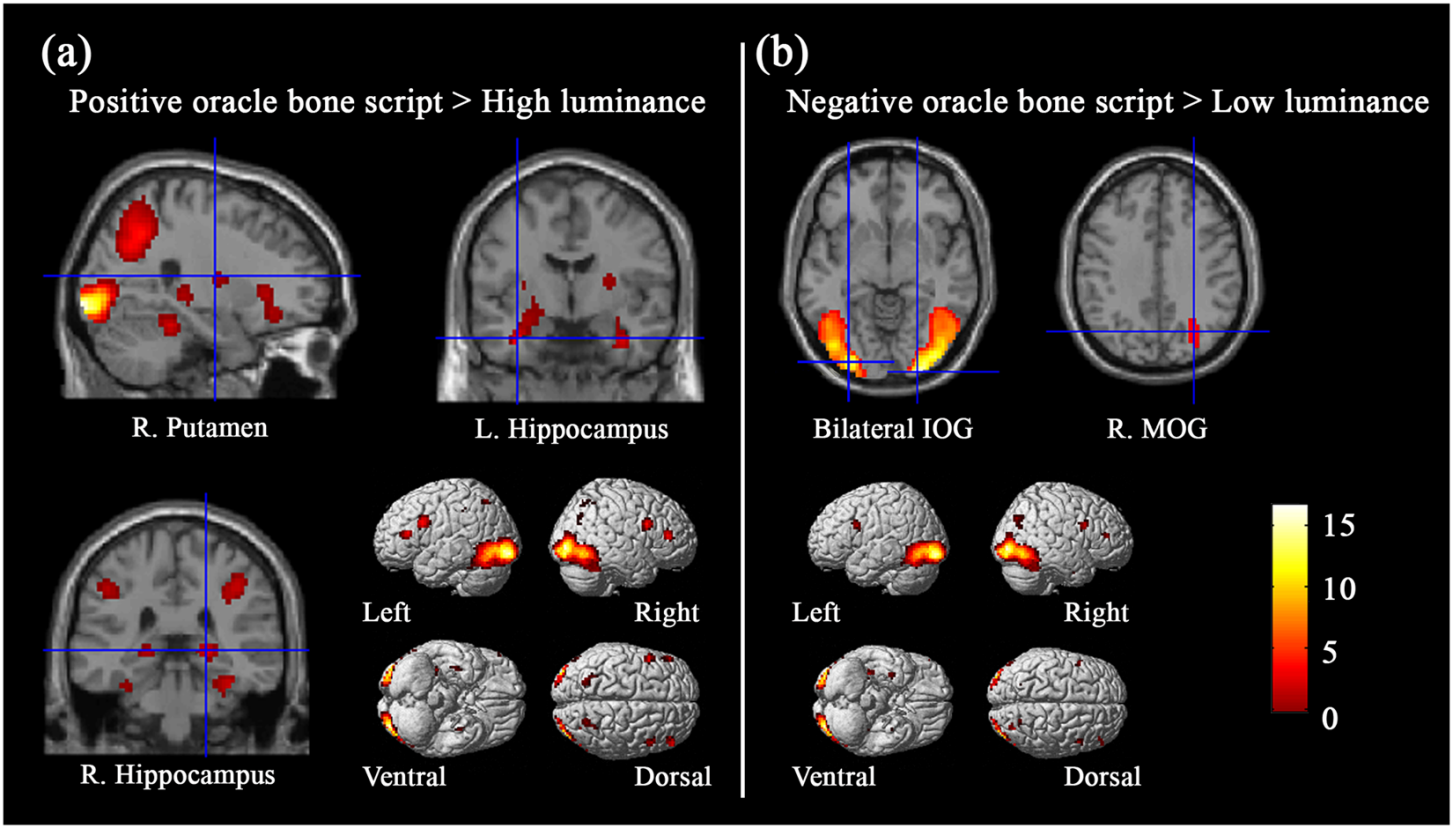
The main cerebral areas for aesthetic judgments of oracle bone scripts.

In the contrast of “OP > HL”, we found that beauty judgments of oracle bone scripts referring to positive social meanings were associated with activity in the bilateral inferior occipital gyri and inferior frontal gyri, left medial superior frontal gyrus, bilateral hippocampus, and right putamen (see Table 2, Fig. 2a). For the contrast of “ON > LL”, we observed significantly stronger activations in the bilateral inferior occipital gyri and right middle occipital gyrus (see Table 2, Fig. 2b).

**Table 2:** Activated areas correlating with the aesthetic judgments of oracle bone scripts.

Brain regions	Hemisphere	Peak coordinates			*t*-score	Cluster size
		x	y	z		
**Positive oracle bone script > High luminance**						
Inferior occipital gyrus	L	−33	−90	−9	18.60	2046
	R	24	−99	−6	18.30	2270
Inferior frontal gyrus	L	−48	12	30	7.09	778
	R	45	12	27	8.16	756
Medial superior frontal gyrus	L	−6	21	42	5.18	269
Hippocampus	L	−33	−6	−27	5.45	234
	R	21	−33	0	5.39	192
Putamen	R	27	−6	12	4.02	21
**Negative oracle bone script > Low luminance**						
Inferior occipital gyrus	L	−33	−90	−9	15.89	933
	R	24	−99	−6	16.58	1000
Middle occipital gyrus	R	30	−66	36	6.50	132

Note: Coordinates refer to the stereotactic space of the Montreal Neurological Institute. The statistical significance refers to *p* < 0.001 at voxel level (uncorrected), *p* < 0.05 at cluster level (FWE corrected).

#### Brain regions revealed by conjunction analysis

**Figure 3:**
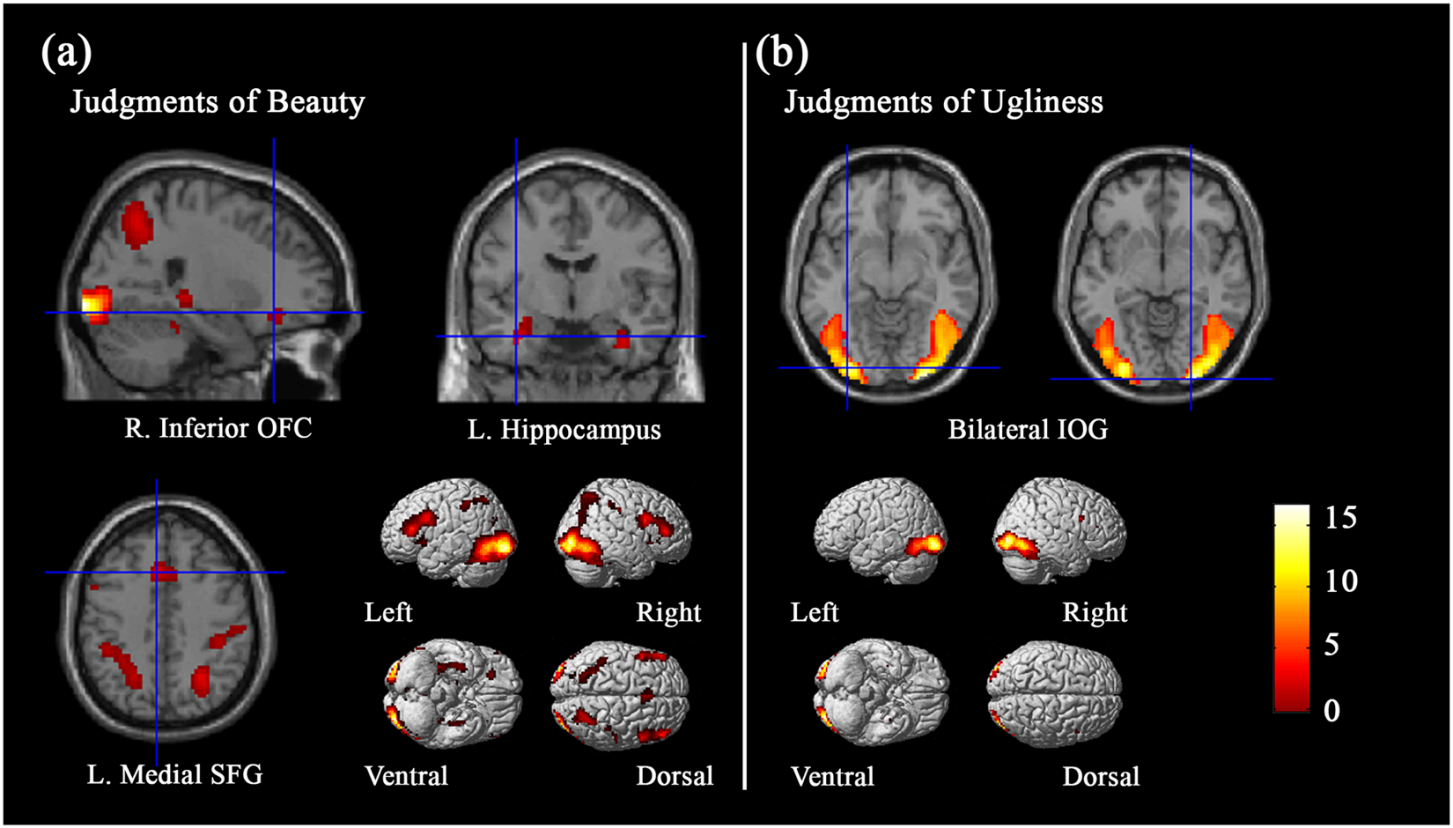
The conjunction results of the judgments of beautiful items and ugly items across pictographs and oracle bone scripts.

Conjunction analyses were performed to identify brain activations common to aesthetic judgments of pictographs and oracle bone scripts. Results showed that bilateral inferior occipital gyri, left middle occipital gyrus, bilateral inferior frontal gyri, left medial superior frontal gyrus, left hippocampus and right inferior OFC were commonly activated when making beauty judgments for both pictographs and oracle bone scripts (see Table 3 and Fig. 3a). Moreover, conjunction analysis revealed common activation of bilateral inferior occipital gyri during judgments of both pictographs that referred to ugly objects and oracle bone scripts that referred to negative social meanings (see Table 3 and Fig. 3b).

**Table 3:** Activations in the conjunction analysis between pictographs and oracle bone scripts.

Brain regions	Hemisphere	Peak coordinates			*t*-score	Cluster size
		x	y	z		
**Conjunction of beautiful pictograph and positive oracle bone script**						
Inferior occipital gyrus	L	−33	−90	−9	17.70	1398
	R	24	−99	−6	18.30	1957
Middle occipital gyrus	L	−24	−72	30	5.73	356
Inferior frontal gyrus	L	−48	9	30	6.78	676
	R	45	12	27	7.27	563
Medial superior frontal gyrus	L	−6	21	42	5.26	160
Hippocampus	L	−33	−6	−27	5.92	61
Inferior OFC	R	27	30	−12	4.42	34
**Conjunction of ugly pictograph and negative oracle bone script**						
Inferior occipital gyrus	L	−33	−90	−9	13.59	638
	R	24	−99	−6	14.24	762

Note: Coordinates refer to the stereotactic space of the Montreal Neurological Institute. The statistical significance refers to *p* < 0.001 at voxel level (uncorrected), *p* < 0.05 at cluster level (FWE corrected).

#### Aesthetic cortical differentiation between pictographs and oracle bone scripts

In order to investigate the cortical differentiation of the aesthetic judgments between pictographs and oracle bone scripts, direct pairwise comparisons were made between the four sets of experimental materials within valence. We found enhanced activation of right cuneus and left inferior temporal gyrus in the contrast of “PB > OP”, but no significant differences were found in the opposite contrast. For the comparisons between pictographs referred to ugly objects and oracle bone scripts referred to negative social meanings, only the contrast of “ON > PU” revealed stronger activations in left superior temporal gyrus and bilateral fusiform gyri, but there was no significant difference in the opposite contrast of “PU > ON” (see Table 4).

**Table 4:** Cortical networks of the analysis of variance between pictographs and oracle bone scripts.

Brain regions	Hemisphere	Peak coordinates			*t*-score	Cluster size
		x	y	z		
**Beautiful pictograph > Positive oracle bone script**						
Cuneus	R	12	−93	−18	4.52	288
Inferior temporal gyrus	L	−60	−24	−18	4.05	50
**Positive oracle bone script > Beautiful pictograph**						
Non-significant						
**Ugly pictograph > Negative oracle bone script**						
Non-significant						
**Negative oracle bone script > Ugly pictograph**						
Superior temporal gyrus	L	−57	−3	−6	4.20	72
Fusiform gyrus	L	−27	−54	−9	4.18	39
	R	33	−39	−15	4.04	103

Note: Coordinates refer to the stereotactic space of the Montreal Neurological Institute. The statistical significance refers to *p* < 0.001 at voxel level (uncorrected), *p* < 0.05 at cluster level (FWE corrected).

#### Cortical differentiation between beautiful and ugly stimuli

The contrast between pictographs that referred to beautiful objects and those that referred to ugly objects revealed stronger activations for pictographs referred to beautiful objects (PB > PU) in the left inferior temporal gyrus and cuneus, right calcarine sulcus and lingual gyrus. The opposite contrast of “PU > PB” revealed no significant activations. For the oracle bone scripts, no significant activations were found in the contrast of “OP > ON”, but stronger activations in the opposite contrast of “ON > OP” were observed in bilateral lingual gyri, left fusiform gyrus, right precentral gyrus, left middle temporal gyrus and paracentral lobule (see Table 5).

**Table 5:** Cortical networks of the analysis of variance between beautiful and ugly stimuli.

Brain regions	Hemisphere	Peak coordinates			*t*-score	Cluster size
		x	y	z		
**Beautiful pictograph > Ugly pictograph**						
Inferior temporal gyrus	L	−57	−54	−15	4.60	48
Cuneus	L	0	−90	18	3.59	48
Calcarine sulcus	R	12	−66	15	4.57	169
Lingual gyrus	R	18	−99	−6	4.41	36
**Ugly pictograph > Beautiful pictograph**						
Non-significant						
**Positive oracle bone script > Negative oracle bone script**						
Non-significant						
**Negative oracle bone script > Positive oracle bone script**						
Lingual gyrus	L	−27	−51	−6	4.43	34
	R	21	−72	−6	3.78	29
Fusiform gyrus	L	−27	−75	−6	3.76	34
Precentral gyrus	R	12	−21	75	4.34	20
Middle temporal gyrus	L	−51	6	−18	4.14	34
Paracentral lobule	L	−6	−33	57	4.00	21

Note: Coordinates refer to the stereotactic space of the Montreal Neurological Institute. The statistical significance refers to *p* < 0.001 at voxel level (uncorrected), *p* < 0.05 at cluster level (FWE corrected).

#### Brain regions for aesthetic judgments regardless of the aesthetic valence

**Figure 4:**
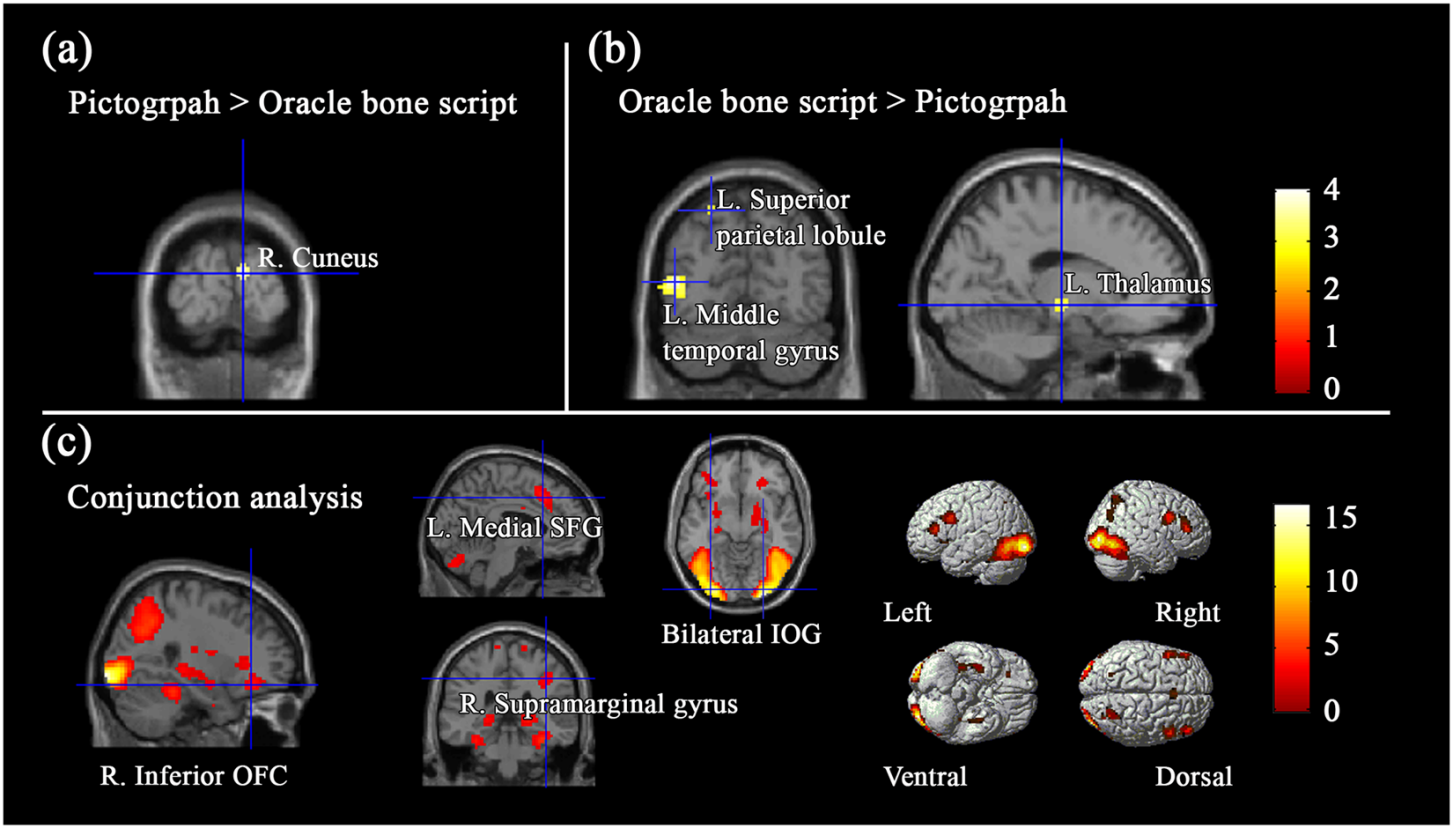
Cerebral areas for aesthetic judgments regardless of the aesthetic valence.

To further understand the brain regions involved in aesthetic processing of concrete pictographs and abstract oracle bone scripts per se, we analyzed fMRI activity in the comparisons for the aesthetic judgment vs. baseline condition. Contrast of “AP > SL” revealed significant activation in the bilateral inferior occipital gyri, left medial superior frontal gyrus, right inferior OFC, left hippocampus, right middle cingulum, right supramarginal gyrus, and right paracentral lobule (see Table 6). In the contrast of “AO > SL”, we found stronger activations in the bilateral inferior occipital gyri, bilateral inferior frontal gyri, left medial superior frontal gyrus, right inferior OFC, and left hippocampus (see Table 6). Meanwhile, we also computed a conjunction analyses between the “AP > SL” and “AO > SL”. The results showed that bilateral inferior occipital gyri, left medial superior frontal gyrus, right middle cingulum, left hippocampus, right inferior OFC, and right supramarginal gyrus were commonly activated (see Table 7 and Fig. 4c).

**Table 6:** Brain regions for aesthetic judgments of pictographs and oracle bone scripts regardless of the aesthetic valence.

Brain regions	Hemisphere	Peak coordinates			*t*-score	Cluster size
		x	y	z		
**Aesthetic judgments of pictograph > Luminance judgments**						
Inferior occipital gyrus	L	−33	−90	−9	21.98	3289
	R	24	−99	−6	23.68	2702
Medial superior frontal gyrus	L	−6	21	42	6.15	393
Inferior OFC	R	27	33	−18	9.15	892
Hippocampus	L	−21	−33	0	5.04	68
Middle cingulum	R	6	0	32	4.79	37
Supramarginal gyrus	R	39	−33	42	4.71	80
Paracentral lobule	R	6	−24	75	4.49	33
**Aesthetic judgments of oracle bone script > Luminance judgments**						
Inferior occipital gyrus	L	−33	−90	−9	24.38	1631
	R	24	−99	−6	24.65	1932
Inferior frontal gyrus	L	−48	9	30	8.71	457
	R	45	12	27	10.12	220
Medial superior frontal gyrus	L	−6	21	42	6.88	137
Inferior OFC	R	27	30	−12	5.77	21
Hippocampus	L	−33	−6	−27	7.74	60

Note: Coordinates refer to the stereotactic space of the Montreal Neurological Institute. The statistical significance refers to *p* < 0.001 at voxel level (uncorrected), *p* < 0.05 at cluster level (FWE corrected).

**Table 7:** Cortical networks of the analysis of conjunction, and the analysis of variance between pictographs and oracle bone scripts regardless of the aesthetic valence.

Brain regions	Hemisphere	Peak coordinates			*t*-score	Cluster size
		x	y	z		
**Conjunction of “AP > SL” and “AO > SL”**						
Inferior occipital gyrus	L	−33	−90	−9	21.98	3105
	R	24	−99	−6	23.68	2613
Medial superior frontal gyrus	L	−6	21	42	6.15	303
Middle cingulum	R	6	0	32	4.79	32
Hippocampus	L	−21	−33	0	5.04	61
Inferior OFC	R	27	30	−15	5.69	850
Supramarginal gyrus	R	39	−33	42	4.71	79
**Aesthetic judgments of pictograph > Aesthetic judgments of oracle bone script**						
Cuneus	R	12	−93	15	4.04	13
**Aesthetic judgments of oracle bone script > Aesthetic judgments of pictograph**						
Middle temporal gyrus	L	−48	−69	9	4.61	96
Superior parietal lobule	L	−24	−69	60	3.52	20
Thalamus	L	−15	−15	−6	3.83	21

Note: Coordinates refer to the stereotactic space of the Montreal Neurological Institute. The statistical significance refers to *p* < 0.001 at voxel level (uncorrected), *p* < 0.05 at cluster level (FWE corrected).

To identify regions sensitive to the differences between pictographs and oracle bone scripts, we conducted two contrasts between conditions “AP” and “AO”. In the contrast of “AP > AO”, we found only right cuneus was activated (see Table 7 and Fig. 4a). Stronger activations were found in the left middle temporal gyrus, left superior parietal lobule, and left thalamus in the opposite contrast of “AO > AP” (see Table 7 and Fig. 4b).

## Discussion

In this present study, we identified the neural basis underlying the aesthetic appraisal of ancient Chinese characters differing in referent (concrete objects versus abstract social concepts) and valence (positive and negative). As hypothesized, the behavioral data indicated that pictographs referring to beautiful concrete objects and oracle bone scripts referring to positive social meanings were more likely to be judged as beautiful. In contrast, pictographs referring to ugly objects and oracle bone scripts referring to negative social meanings were more likely to be judged as ugly, showing that the experimental materials used in the present study are capable of inducing aesthetic experience and appraisal.

In line with previous findings, the bilateral inferior occipital gyri, bilateral inferior frontal gyri, left medial superior frontal gyrus, left hippocampus and right inferior OFC were activated during the aesthetic judgments (analysis regardless of aesthetic valence) and beautiful font structure judgments (analysis with aesthetic valence) of pictographs^21^. Prior findings have indicated that occipital gyrus plays an important part in early visual processing such as perception of shape and color^30^, as well as visual attention to the beautiful visual stimuli^21, 31, 32^. The inferior frontal gyrus is also active in the aesthetic judgments of rhetorical beauty and moral beauty^33^, and this activation is considered to represent emotional states regardless of the actual affective or sensory modality^34^. Meanwhile, the superior frontal gyrus is related to aesthetic judgment and social cognition^5, 35^, and the hippocampus is known for a functional correlation between processing of novel stimuli and evaluation of aesthetic and pleasant stimuli^36–39^. The OFC has been suggested to be important for aesthetic perception and judgment, and is reliably activated during both aesthetic appraisal and non-aesthetic appraisal tasks^19, 40^. Previous studies also proposed that the OFC plays a key role in emotion, and is involved in the functional integration of emotion and cognition^41, 42^, which may be present in aesthetic emotion^40^.

More importantly, previous neuroimaging evidence suggests the sense of beauty may heighten spatial cognitive processes, increase somatosensory perception, and elicit the planning and intention of movements^43^. Our results support prior findings in which stronger activation of superior parietal lobule, right supramarginal gyrus extending to postcentral gyrus, and right paracentral lobule were found during the aesthetic judgments of pictographs. For instance, the superior parietal lobule is related to the visuo-spatial exploration and motor mapping^32, 44^, supramarginal gyrus extending to postcentral gyrus is part of somatosensory association cortex^45, 46^, and paracentral lobule is concerned with motor function and is often called the supplementary motor area^47–49^. Since we adopted the square luminance judgment task as a baseline task to control for the activation of motor-related areas in response to key pressing, we infer that visuo-spatial exploration and motor representation may be associated with aesthetic orientation. More precisely, these motor-related regions may be implicated in the generation of embodied approach and motivation for beauty, and the above neural results could be interpreted as indicating that beauty judgments of pictographs involve joint participation of perceptual processing, cognitive judgment, reward and embodied experience^21, 28, 29^.

For both the aesthetic judgments overall and beautiful judgments specifically of oracle bone scripts, neuroimaging results revealed similar neural regions including bilateral inferior occipital gyri, bilateral inferior frontal gyri, left medial superior frontal gyrus, and bilateral hippocampus. We also found the right inferior OFC was active regardless of aesthetic valence, and the right putamen was active for beauty judgments of oracle bone scripts that referred to positive social meanings. Although no activation in the inferior OFC was found in the analysis with aesthetic valence, the putamen has been strongly implicated in reward process^19, 50–53^. As hypothesized, our results are consistent with previous neuroaesthetics findings, and could be interpreted as that beautiful judgments of oracle bone scripts referring to positive social meanings also commonly rely on perceptual, cognitive, emotional and reward processes. More importantly, the involvement of these regions clearly indicates that aesthetic appraisals of concrete pictographs and abstract oracle bone scripts alike, are not simple reactions, but rather involve multiple cognitive functions with widespread distributed neural networks^30^.

In contrast to judgments of beauty for pictographs, no activity in motor brain networks related to embodied approaching motivation was found when making judgments of beauty for oracle bone scripts. We propose that this absence of activation of motor areas may be attributed to low visualization demands of the oracle bone script stimuli. More specifically, pictographs were generated by outlining the external morphologies of their referential objects^22, 23^, making them more similar to their referential objects and having higher visualization demands than abstract oracle bone script stimuli. Hence, more perceptual representations were activated because of high visual imagery of pictographs, which facilitated the generation of embodied experience.

It is likely that only perceptual processing was engaged when deciding that pictographs and oracle bone scripts were ugly or negative; our conjunction analysis revealed common activations in bilateral inferior occipital gyri. This result replicated similar findings from studies using graphic patterns^54^ and pictographs^21^ as materials which were judged as not-beautiful or ugly, suggesting the ugly materials can be regarded as aversive stimuli^28^ and have an inhibitory effect on further cognitive and emotional processing.

Greater activation in the cuneus and inferior temporal gyrus was observed for pictographs than for oracle bone scripts during judgments of beauty. However, no significant activation was found in the opposite contrast. Previous studies have related the inferior temporal gyrus to visual imagery^55^ and the representation of object shape^56^, and the cuneus to the aesthetic appreciation of beauty^32, 57^. Thus, the stronger activation in these brain structures may be explained by a tendency for higher aesthetic preference for stimuli with high visualization demands^10, 58^. During judgments of ugly pictographs and negative oracle bone scripts, only the contrast of “negative oracle bone script > ugly pictograph” resulted in significant activation differences, which were located in the superior temporal gyrus and fusiform gyrus. Previous studies have demonstrated that the superior temporal gyrus is involved in a more abstract aesthetic processing^59^ (e.g., moral beauty), and the fusiform gyrus is related to perceptual processing^32, 37^. Therefore, it is possible that activity in these brain regions served as an extra neural resource for the aesthetic judgments of oracle bone script due to their low visualization.

To summarize, the present findings support our hypothesis that pictographs and oracle bone scripts can elicit aesthetic appraisal related to their referential objects and social meanings, respectively. Neural correlates of aesthetic appraisal of the two sets of materials suggest that judgments of beauty for pictographs and oracle bone scripts rely on common neural pathways supporting perceptual, cognitive and reward processing. Moreover, only judgments of beauty for pictographs referring to concrete objects, but not oracle bone scripts referring to abstract social meanings involved motor-related circuitry, which implies higher visualization is associated with embodied experience and approaching motivation for beauty.

Theoretically, our behavioral and neuroimaging findings provide preliminary interpretations for the mental and neural mechanisms of the two exclusive processes of beauty by using ancient Chinese characters, which showed that the aesthetic judgments of pictographs mainly depended on the aesthetic qualities of the referential objects, and involved activity in reward and motor-related areas. It indicates that beauty originates from the objective parameters and morphologies of stimuli, which can be regarded as the sense of embodied natural beauty and support the theory that “beauty has its corresponding morphological characteristics”. For oracle bone scripts, the aesthetic judgments were affected by the positive degree of their social meanings, and the corresponding judgments of beauty additionally activated subcortical reward regions. This indicates that beauty is also affected by subjective social construction, which can be regarded as the sense of social endowed beauty and is in accordance with the theory “what is good is beautiful, and bad is ugly”.

## Methods

### Participants

Nineteen healthy right-handed college students (12 females) between 19 to 27 years of age (mean age = 21.74, *SD* = 2.18) gave written informed consent for this experiment, according to procedures approved by the Institute Ethics Committee, South China Normal University. None of them had received university education within any Chinese language-related majors or professional training in the arts. All participants had normal or corrected-to-normal vision, and none of them had a history of neurological or psychiatric disorders. They each received 100 yuan RMB (about $15) for their participation. The study was design and conducted in accordance with the guidelines of the Helsinki Declaration.

### Experimental procedures

#### Stimuli

**Figure 5:**
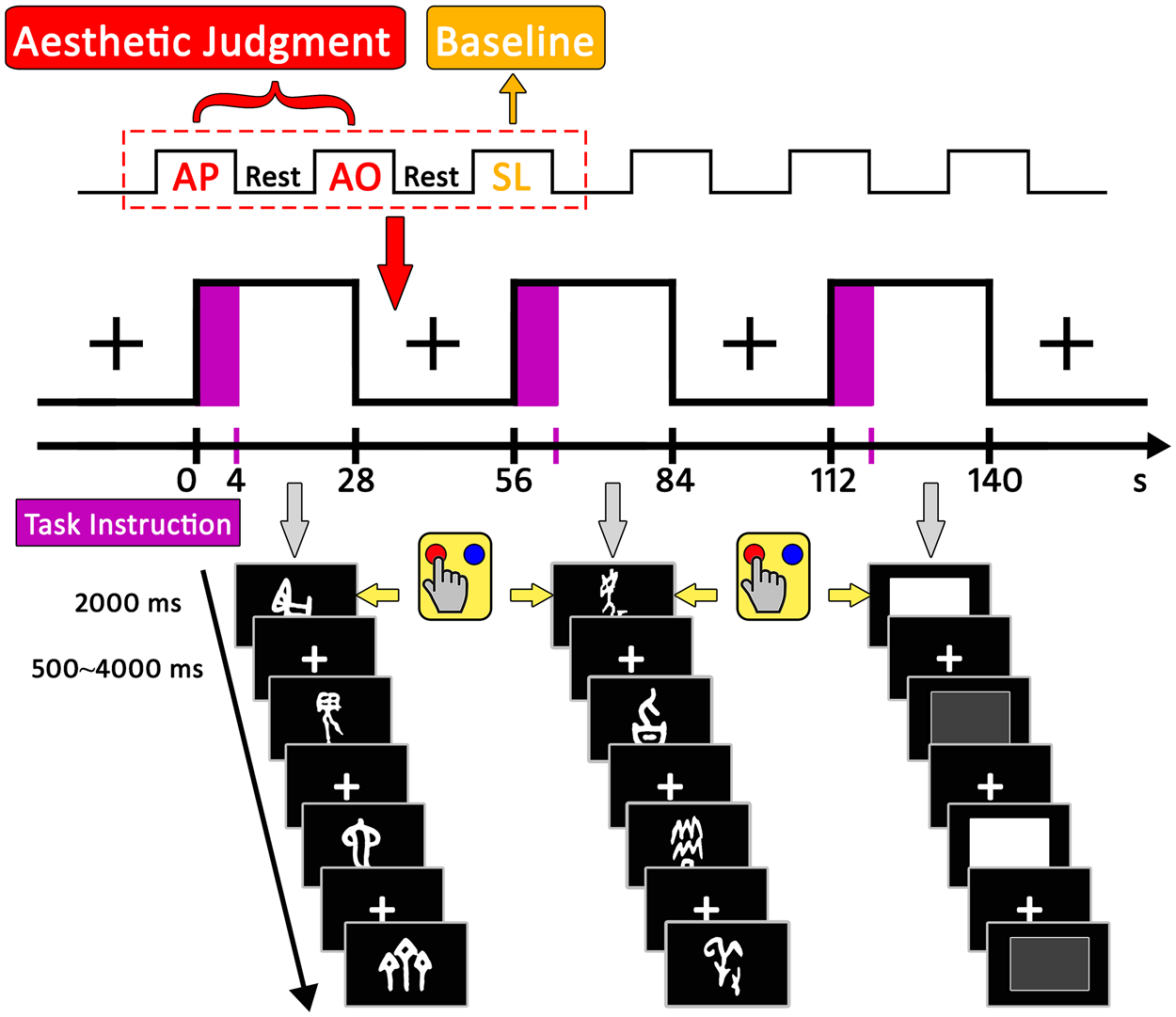
Experimental design, procedure and examples of stimuli. Three types of tasks were performed in separate blocks: AP judgments (beautiful pictograph vs. ugly pictograph), AO judgments (positive oracle bone script vs. negative oracle bone script), SL judgments (high luminance vs. low luminance). Examples in the first column are pictographs, referring to “rabbit”, “sacrificial mask”, “boa constrictor” and “seedling”, from top to bottom. Examples in the second column are abstract oracle bone scripts, referring to “contemptuous”, “sweet”, “catastrophic” and “courteous”, from top to bottom. Examples in the third column are grey squares, for high and low luminance, respectively.

The samples of stimuli and the experimental procedures for the present study are illustrated in Fig. 5. We used three categories of stimuli: pictographs, abstract oracle bone scripts, and grey squares. Pictographs are ancient Chinese characters that referred to concrete objects, which were adopted from the study by Zhang *et al*.^20, 21^.

Abstract oracle bone scripts are ancient Chinese characters that referred to abstract social meanings. The oracle bone script meanings were based on the semantic interpretation provided by *Oracle Bone Scripts Dictionary* ^60^ and were rated by a separate group of participants (*n* = 23) as to whether they reflected positive social meaning and negative social meaning. From an original pool of 98 abstract oracle bone scripts, we selected 24 that referred to positive social meanings and 24 that referred to negative social meaning. The first set of ratings presented the modern simplified two-character Chinese words corresponding each of the 98 abstract oracle bone scripts. For each word, participants rated its social meaning (positive or negative) on a 7-point scale, with 1 for extremely negative, and 7 for extremely positive. Ratings of the positive degrees of their social meanings between the two sets of experimental materials (6.24 ± 0.35; 1.86 ± 0.49, for positive and negative social meanings that the words referred to, respectively) were significant different, *F* (1, 46) = 1277.09, *p* < 0.001.

The second set of ratings used the 98 abstract oracle bone scripts as materials. The easiness of guessing the semantic meaning of oracle bone script and the visual complexity of oracle bone script were also rated on a scale from 1 to 7, 1 for extremely hard or extremely simple, and 7 for extremely easy or extremely complicated. The rating results showed no significant differences in terms of the easiness of guessing the semantic meanings of oracle bone scripts (1.53 ± 0.29; 1.37 ± 0.39, for oracle bone scripts referring to positive and negative social meanings, respectively), *F* (1, 46) = 2.59, *p* = 0.115. There were also no significant differences in terms of the complexity (4.36 ± 0.79; 4.71 ± 0.95, for oracle bone scripts referring to positive and negative social meanings, respectively), *F* (1, 46) = 1.96, *p* = 0.168.

For grey squares, we adopted 24 high luminance grey squares (RGB = 255, 255, 255) and 24 low luminance grey squares (RGB = 64, 64, 64) from the study by Zhang *et al*.^21^.

The pictographs and abstract oracle bone scripts were presented in a 100-point white font. Grey squares were adjusted to be equal in size within a rectangular ‘window’ sized 300 × 200 pixels. All the three types of experimental stimuli were centered on a 600 × 400 pixel black background and presented at a screen resolution of 800 × 600 pixels.

#### Task

Participants performed two kinds of tasks during the scanning. In the aesthetic judgment task, participants were instructed to judge if the pictograph (block AP) or oracle bone script character (block AO) was beautiful or ugly by pressing one of two buttons with different hands. The square luminance judgment task served as a baseline task, similar to the studies by Tsukiura & Cabeza^18^ and Zhang *et al*.^21^. Participants were instructed to judge whether the luminance of the square (block SL) was high or low by pressing one of two buttons. The finger-response mapping was counterbalanced across participants.

In order to avoid participants knowing or being familiar with the semantic meaning of stimuli, each participant rated the easiness of guessing the semantic meanings of pictographs and oracle bone scripts which were used in the experiment outside the MR-scanner by using a 7-point scale, with 1 for very hard, and 7 for very easy.

#### Procedure

The scanning session used a hybrid design with 16 blocks pertaining of each of the three conditions, AP, AO, and SL. Each condition included 48 stimuli each repeated once, resulting in a total of 96 trials. Participants underwent 4 separate scanner runs; each run consisted of 12 blocks. Block order was fixed and counterbalanced across participants. Each block contained 6 trials and lasted for 28 s. There was a 28 s fixation interval between blocks. At the beginning of each block, a 4 s visual task instruction informed the participants about task to be performed in the upcoming block. On each trial the stimulus was presented in the center of the screen for 2 s including response time in pseudo-random order (event-related design), and was followed by a jittered 500–4000 ms inter-stimulus interval (ISI).

### Data acquisition

Anatomical T1-weighted and functional T2*-weight MR images were conducted on a 3-Tesla Siemens Trio Tim MRI scanner, using a 12-channel phased-array head coil at the Magnetic Resonance Imaging Lab, South China Normal University. Functional images were acquired using a T2*-weighted gradient echo, echo-planar imaging sequence (32 axial slices covering the whole brain, TR = 2000 ms, TE = 30 ms, flip angle = 90°, FOV = 192 mm, inter-slice gap = 1 mm, slice thickness = 3 mm, matrix size = 64 × 64). After the functional scanning, a high-resolution T1-weighted anatomical scan was acquired by using a MP-RAGE sequence (TR = 1900 ms, TE = 2.52 ms, flip angle = 9°, voxel-size = 1 mm × 1 mm × 1 mm).

### Data analysis

Image pre-processing and analysis were performed using SPM8 (http://www.fil.ion.ucl.ac.uk/spm/). The first 6 volumes of each functional run were discarded to allow for T1 equilibration. All remaining volumes from each participant were preprocessed with slice-scan time correction, spatially realigned to the first volume for correcting head movements, coregistered to the T1-weighted structural image, normalized to the standard template based on the MNI reference brain, resampled with voxel size of 3 × 3 × 3 mm^3^, and spatially smoothed with an isotropic 8 mm full width-half-maximum (FWHM) Gaussian kernel. Two participants were excluded in the subsequent analysis as one individual image had >2 mm maximum displacement and >1.5° rotation, and one individual had low accuracy rates for the aesthetic judgments.

At the first (single participant) level, a general linear model (GLM) was applied to the time-series data, in which stimulus onset was modeled as a single impulse response function, and then convolved with the canonical haemodynamic response function (HRF). We modeled six regressors of interest: pictograph referring to beautiful object (PB), pictograph referring to ugly object (PU), oracle bone script referring to positive social meaning (OP), oracle bone script referring to negative social meaning (ON), high luminance (HL) and low luminance (LL). Head movement parameters calculated from the realignment procedure were included as regressors of no interest. Low frequency drifts were removed using a high-pass filter with a cut-off of 128 s. The resulting contrast images for each condition were entered into the second level (random-effects) analyses in which the regressors of interest were modeled using Flexible Factorial analyses. In order to identify the cortical networks involved in the aesthetic judgments of pictographs and oracle bone scripts we utilized the square luminance judgment as baseline to control for activity in motor brain regions associated with the key responses. Therefore, we first performed the contrasts of “PB > HL”, “PU > LL”, “OP > HL” and “ON > LL”. Based on the four contrasts, we computed a conjunction between the “PB > HL” and “OP > HL”, “PU > LL” and “ON > LL” using the minimum statistic approach^61^. Moreover, direct comparison of “PB > OP”, “PU > ON”, “OP > PB” and “ON > PU” were conducted to investigate differences in neural mechanisms between aesthetic judgments of pictographs and oracle bone scripts. Statistical thresholds for Flexible Factorial analyses were set at *p* < 0.001 at voxel level (uncorrected), *p* < 0.05 at cluster level (FWE corrected).
